# Interaction between occupational physical burdens and low job control on musculoskeletal pain: Analysis of the 5th Korean Working Environment Survey

**DOI:** 10.1002/1348-9585.12244

**Published:** 2021-07-01

**Authors:** Jongin Lee, Hyoung‐Ryoul Kim, Dong‐Wook Lee, Mo‐Yeol Kang

**Affiliations:** ^1^ Department of Occupational and Environmental Medicine Seoul St. Mary’s Hospital College of Medicine The Catholic University of Korea Seoul Republic of Korea; ^2^ Department of Preventive Medicine College of Medicine Seoul National University Seoul Republic of Korea

**Keywords:** ergonomics, interaction, job control, musculoskeletal pain, workplace

## Abstract

**Objective:**

This study aimed to investigate the interactive impacts between occupational physical burdens and psychological job demand or control on musculoskeletal pain (MSP) using nationally representative data for Korean workers.

**Methods:**

Using 5th Korean Working Conditions Survey (KWCS), we explored the interaction between occupational physical burdens and levels of psychological job demand or control on risk of MSP in 49 572 eligible participants. For quantitative evaluation of the interaction, relative excess risk due to interaction (RERI) was calculated.

**Results:**

In a group with low job control and at least one occupational physical burden, odds ratio (OR) for neck and upper extremity pain was 2.44 (95% CI, 2.24‐2.66) compared with a group with high job control and no physical burden (a reference group: lowest risk), which was the highest value among the four groups, and the RERI was 0.35 (95% CI, 0.19‐0.51). Similarly, OR for lower extremity pain was 2.15 (95% CI, 1.95‐2.37) and RERI was 0.26 (95% CI, 0.07‐0.45). However, the RERI was not significant in the case of psychological job demand.

**Conclusion:**

This study revealed significant interactions between occupational physical burdens and low job control on MSP.

## 1 INTRODUCTION

Work‐related musculoskeletal disorders are cumulative traumatic diseases caused by occupational physical burdens; although multidimensional factors affect its risk. In 1997, the National Institute of Occupational Safety and Health suggested various occupational physical burdens as the cause of work‐related musculoskeletal disorders. These factors include awkward posture, overhead work, carrying objects in a twisted posture, bending of the wrist, contact stress, poor posture of the shoulder and wrist, handling of heavy loads, use of vibratory tools, and whole‐body vibration.^1^ In addition to these mechanical and direct occupational physical burdens that cause repeated accumulation of damage, other potential risk factors for musculoskeletal disorders include genetic backgrounds,^2^ chronic diseases,^3^ and working environments,^4^ such as low temperatures and insufficient rest. Moreover, psychological factors also have an important role in musculoskeletal pains or disorders.^5^ For example, job demand, control over job‐related decisions, monotony, job satisfaction, supervisor and co‐worker support, and work pace have been demonstrated to have significant associations with the risk of musculoskeletal disorders.^6^ Furthermore, decrease in job satisfaction, stress responses, social relations, and emotional labor are linked to various musculoskeletal disorders.^7^

In many cases, physical and psychological factors may work together. Even when workers have a similar burden from occupational physical burdens, it is expected that there will be a difference in the development of musculoskeletal disorders due to different psychological factors. For example, a job requiring high level of physical burdens in combination with psychological stress, such as an unfair order from a boss, may further increase the risk of musculoskeletal disorders. Moreover, occupational physical burdens sometimes increase the perception of psychological stress. Therefore, psychological stress and physical burdens might have a synergistic effect on the development and progression of musculoskeletal disorders. Consistently, Widanarko et al revealed interactive impacts between psychological factors and occupational physical burdens on musculoskeletal disorders in the neck, upper extremities, and lower back among Indonesian coal mine workers.^8^, ^9^ However, these two studies, published by the same researchers, included only coal mine workers with heavy physical work. To date, a few studies have investigated musculoskeletal disorders in various types of occupations, including jobs with less labor‐intensive tasks and more complex psychological factors.^10^

Hence, it could conceivably be hypothesized that the major determinants of job strain, psychological job demand, and control may have interactive relationships with occupational physical burdens on musculoskeletal pain (MSP). This study aimed to investigate the interactive relationship between occupational physical burdens and psychological job demand or control on the risk of MSP using nationally representative data for Korean workers.

## 2 METHODS

### 2.1 Study participants

A total of 50 205 participants were selected for our study from the 5th Korean Working Conditions Survey (KWCS). The KWCS, conducted by the Korea Occupational Safety and Health Agency, has a similar structure to and licensed the same survey items as the European Working Conditions Survey.^11^ The survey target was the working population over 15 years old, and the study participants consisted of waged workers, unpaid family workers, self‐employed workers, and employers who run their own businesses. Participants with missing values were excluded from each analysis step, and finally, 49 572 (98.7%) participants were included in the analysis. This study basically included all types of workers: waged workers were used for sensitivity analysis.

### 2.2 Definition of variables

Research items for various physical burden factors, including “awkward posture,” “lifting or moving a person,” “dragging, pushing, or moving heavy objects,” and “repetitive hand or arm movements,” constituted a subcategory of the working conditions. The responses to each subitem are as follows: “1. All working hours,” “2. Almost all working hours,” “3. 3/4 of working hours,” “4. Half of working hours,” “5. 1/4 of working hours,” “6. Hardly exposed,” and “7. Never exposed.” The Korean law defines “musculoskeletal burden tasks” as conducting more than 2 hours of physical burden factors listed in the study questionnaire items. However, considering self‐report questions and distribution of the responses, participants who answered that at least one sub‐item took more than 3/4 of working hours were assigned as the corresponding group for physical burden factors.

Job control was assessed by the following three questions: “I can take a break when I want,” “I can apply my thoughts to my work,” and “I can influence important decisions in the business.” For each of these questions, the responses are as follows: “1. Always,” “2. Most of the time,” “3. Sometimes,” “4. Not really,” and “5. Not at all.” In response, the numbers of the relevant items were regarded as the score for the four questions and were summed up into one value. The participants who had lower than median values (10 points) were classified as the low job control group.

Psychological job demand was assessed by the following four questions: “I work very fast at work,” “I have to work on time strictly,” “I have enough time to finish my work,” and “I get stress from my work.” The responses and methods for classification of the participants were the same as the questions above for job control. Because the third questionnaire (I have enough time to finish my work) had an opposite direction compared with the other items, we used inverse scores to the item.

Musculoskeletal symptoms, the dependent variable of this study, were investigated using the following question: “Over the last 12 months, did you have any of the following health problems?” Symptoms were largely categorized into three groups: “back pain,” “neck and upper extremity pain (shoulder, neck, arms, elbows, wrists, and hands),” and “lower extremity pain (hips, legs, knees, and feet).”

Occupations were classified according to the 6th Korea Standard Classification of Occupation (KSCO) which is a modified version of International Classification of Occupation (ISCO). The wage levels were categorized into three groups: <2 million won (about 1770 US dollar equivalent at the exchange rate of 1130 won/US dollar) per month, 2‐4 million won (about 3540 US dollar equivalent) per month, and more than 4 million won/month. Taking into account the working hours and the upper limit according to the Labor Standards Act, weekly working hours were divided into three groups: ≤40, 41‐52, and >52 hours. Shift work was assessed by a question about the work type: “I work in shifts.” The answer could be “Yes,” “No,” “Do not know,” or “refusal.”

### 2.3 Statistical analysis

The job control and occupational physical burdens were designed as the main explanatory variables in this study. Because psychological job demand and control are often assessed together, we also examined the association between psychological job demand and occupational physical burdens. First, we examined the association of psychological job demand, job control, and occupational physical burdens with demographic variables, including sex, age, education level, monthly wage, job category with chi‐squared test. Prevalence of MSP was also analyzed using chi‐squared test according to the presence of occupational physical burdens and levels of psychological job demand and job control, respectively. In addition, multiple logistic regression analysis was performed to determine whether musculoskeletal symptoms were associated with the presence or absence of occupational physical burdens. Moreover, in order to analyze the interaction between occupational physical burdens and levels of psychological job demand or control, the odds ratios for MSP were calculated based on psychological job demand or control (four categories: low occupational physical burdens and high job control/low psychological job demand as a reference group) using a multiple logistic regression model adjusting for sex, age, education level, monthly wage, job category, weekly working hours, and shiftwork. For quantitative evaluation of the interaction, relative excess risk due to interaction (RERI) was calculated. The RERI from two exposures (A and B) is calculated with the following formula: RERI = ERR (AB) – ERR (A$\bar{B}$) – ERR ($\bar{A}$B), where ERR is an excess relative risk (RR – 1) and $\bar{A}$ means the absence of A. Generally, when the RERI value is a positive number exceeding 0, it is assumed that there is a synergistic interaction.^12^, ^13^ In addition, similar models were used to conduct sensitivity analysis among the subset of waged workers only.

R program version 4.0.3 (Vienna, Austria) was used for statistical analysis, and the R survey package was used to apply weights according to stratified sample extraction during the analysis process. By using the survey weights, the analyses may represent the whole national working population of Korea. Statistical significance was evaluated by 95% confidence intervals and a *P*‐value of 0.05.

## 3 RESULTS

Of all the eligible 49 572 study participants, 16 080 (32.4%) had high psychological job demand, 19 253 (38.8%) had low job control, and 25 261 (50.1%) had at least one occupational physical burden. High psychological demand was more prevalent among those who are men (49.2% vs 46.1% in a high psychological job demand group vs a low group), those with middle monthly wage (48.5% vs 43.5%), craft and related trades workers (12.1% vs 7.1%), and plant and machine operators (12.3% vs 7.1%). With low job control, the following variables showed associations: women (56.7% vs 50.4% in a low job control group vs a high group), low proportion of higher educational level (39.7% vs 44.2%), low monthly wage (49.4% vs 34.8%), elementary workers (16.8% vs 6.2%), short working hours (52.0% vs 46.2%), and shiftwork (12.0% vs 6.3%). Regarding occupational physical burdens, women (53.8% vs 51.9% in a group of at least one occupational physical burdens vs a group without physical burden), older workers (>60, 27.8% vs 21.7%), education level below middle school (42.1% vs 38.8%), low monthly wage (42.1% vs 38.8%), blue‐collar workers such as craft and related trades workers/plant and machine operators/elementary workers (10.6% vs 6.8%/11.9% vs 5.6%/11.9% vs 8.6%), and long working hours (26.5% vs 24.2%) were associated with one or more occupational physical burdens (Table 1).

**TABLE 1:** Demographic characteristics according to psychological job demand, job control, and occupational physical burdens

	Psychological job demand*		Job control*		Occupational physical burdens*	
	Low (N = 33 492)	High (N = 16 080)	High (N = 30 319)	Low (N = 19 253)	No (N = 24 311)	At least one (N = 25 261)
Sex						
Men	15 454 (46.1%)	7919 (49.2%)	15 032 (49.6%)	8341 (43.3%)	11 702 (48.1%)	11 671 (46.2%)
Women	18 038 (53.9%)	8161 (50.8%)	15 287 (50.4%)	10 912 (56.7%)	12 609 (51.9%)	13 590 (53.8%)
Age						
15‐19	161 (0.5%)	94 (0.6%)	79 (0.3%)	176 (0.9%)	127 (0.5%)	128 (0.5%)
20‐29	2658 (7.9%)	1496 (9.3%)	1937 (8.0%)	2217 (11.5%)	2189 (9.0%)	1965 (8.1%)
30‐39	5577 (16.7%)	2820 (17.5%)	4892 (20.1%)	3505 (18.2%)	4403 (18.1%)	3994 (16.5%)
40‐49	7887 (23.5%)	3777 (23.5%)	1388 (5.7%)	4276 (22.2%)	6049 (24.9%)	5615 (23.1%)
50‐59	8497 (25.4%)	4585 (28.5%)	8336 (34.3%)	4746 (24.7%)	6278 (25.8%)	5804 (23.9%)
60‐	8712 (26.0%)	3308 (20.6%)	7687 (31.6%)	4333 (22.5%)	5265 (21.7%)	6755 (27.8%)
Education level						
Below middle school	6867 (20.5%)	2755 (17.1%)	5853 (19.3%)	3769 (19.6%)	3716 (15.3%)	5906 (23.4%)
High school	12 076 (36.1%)	6824 (42.4%)	11057 (36.5%)	7843 (40.7%)	8532 (35.1%)	10368 (41.0%)
Above college	14 549 (43.4%)	6501 (40.4%)	13409 (44.2%)	7641 (39.7%)	12 063 (49.6%)	8987 (35.6%)
Monthly wage (KRW)						
<2000K (USD $1700)	14 088 (42.1%)	5966 (37.1%)	10541 (34.8%)	9513 (49.4%)	9426 (38.8%)	10628 (42.1%)
2000K‐4000K	14 554 (43.5%)	7793 (48.5%)	14227 (46.9%)	8120 (42.2%)	10 889 (44.8%)	11458 (45.4%)
>4000K (USD $3400)	4850 (14.5%)	2321 (14.4%)	5551 (18.3%)	1620 (8.4%)	3996 (16.4%)	3175 (12.6%)
Job category						
Managers	160 (0.5%)	46 (0.3%)	177 (0.6%)	29 (0.2%)	152 (0.6%)	54 (0.2%)
Professionals	5334 (15.9%)	1963 (12.2%)	4799 (15.8%)	2498 (13.0%)	4423 (18.2%)	2874 (11.4%)
Clerical workers	4624 (13.8%)	2065 (12.8%)	3961 (13.1%)	2728 (14.2%)	3836 (15.8%)	2853 (11.3%)
Service workers	4627 (13.8%)	2669 (16.6%)	4452 (14.7%)	2844 (14.8%)	3170 (13.0%)	4126 (16.3%)
Sales workers	6841 (20.4%)	2472 (15.4%)	5946 (19.6%)	3367 (17.5%)	5793 (23.8%)	3520 (13.9%)
Skilled agricultureres	4003 (12.0%)	992 (6.2%)	3990 (13.2%)	1005 (5.2%)	1833 (7.5%)	3162 (12.5%)
Craft and related trades workers	2367 (7.1%)	1948 (12.1%)	2732 (9.0%)	1583 (8.2%)	1648 (6.8%)	2667 (10.6%)
Plant and machine operators	2390 (7.1%)	1973 (12.3%)	2396 (7.9%)	1967 (10.2%)	1365 (5.6%)	2998 (11.9%)
Elementary workers	3146 (9.4%)	1952 (12.1%)	1866 (6.2%)	3232 (16.8%)	2091 (8.6%)	3007 (11.9%)
Weekly working hours						
≤40	16 747 (49.5%)	7279 (45.3%)	14009 (46.2%)	10017 (52.0%)	12067 (49.6%)	11959 (47.3%)
41‐52	8368 (24.7%)	4612 (28.7%)	7733 (25.5%)	5247 (27.3%)	6364 (26.2%)	6616 (26.2%)
>52	8733 (25.8%)	4189 (26.1%)	8577 (28.3%)	3989 (20.7%)	5880 (24.2%)	6686 (26.5%)
Shiftwork						
No	30 894 (92.2%)	14463 (89.9%)	28406 (93.7%)	16951 (88.0%)	22266 (91.6%)	23091 (91.4%)
Yes	2598 (7.8%)	1617 (10.1%)	1913 (6.3%)	2302 (12.0%)	2045 (8.4%)	2170 (8.6%)

* All variable comparisons using chi‐squared tests were statistically significant except for shiftwork and occupational physical burdens (*P* = 0.486).

The prevalence rates of back pain, neck and upper extremity pain, and lower extremity pain were 14.7% (7309 participants), 28.1% (13 922 participants), and 20.6% (10 202 participants), respectively. High psychological job demand was associated with pain on neck and upper extremity (29.4%, *P* < 0.001), however, it showed an inverse association with back pain (*P* < 0.001, higher proportion of back pain (15.4%) with low psychological job demand). Low job control was also associated with back pain inversely (*P* < 0.001, higher proportion of back pain [15.3%] with high job control), but associations between neck and upper extremity pain and low job control, as well as lower extremity pain, were each statistically insignificant (*P* = 0.373 and 0.093, respectively). Presence of at least one occupational physical burden was associated with all types of pains significantly (*P* < 0.001 for all) (Table 2).

**TABLE 2:** Prevalence of back pain, neck and upper extremity pain, and lower extremity pain by occupational physical burdens and job control

	No	Yes	*P* value*
Back pain			
All	42 263 (85.3%)	7309 (14.7%)	
Psychological job demand			
Low	28 336 (84.6%)	5156 (15.4%)	<0.001
High	13 927 (86.6%)	2153 (13.4%)	
Job control			
High	25 680 (84.7%)	4639 (15.3%)	<0.001
Low	16 583 (86.1%)	2670 (13.9%)	
Occupational physical burdens			
No	21 862 (89.9%)	2449 (10.1%)	<0.001
At least one	20 401 (80.8%)	4860 (19.2%)	
Pain on neck and upper extremity			
All	35 650 (71.9%)	13,922 (28.1%)	
Psychological job demand			
Low	24 292 (72.5%)	9200 (27.5%)	<0.001
High	11 358 (70.6%)	4722 (29.4%)	
Job control			
High	21 848 (72.1%)	8471 (27.9%)	0.373
Low	13 802 (71.7%)	5451 (28.3%)	
Occupational physical burdens			
No	19 589 (80.6%)	4722 (19.4%)	<0.001
At least one	16 061 (63.6%)	9200 (36.4%)	
Pain on lower extremity			
All	39 370 (79.4%)	10,202 (20.6%)	
Psychological job demand			
Low	26 548 (79.3%)	6944 (20.7%)	0.228
High	12 822 (79.7%)	3258 (20.3%)	
Job control			
High	24 005 (79.2%)	6314 (20.8%)	0.093
Low	15 365 (79.8%)	3888 (20.2%)	
Occupational physical burdens			
No	20 841 (85.7%)	3470 (14.3%)	<0.001
At least one	18 529 (73.4%)	6732 (26.6%)	

* Chi‐squared test.

Multiple logistic regression models adjusted for sex, age, education level, monthly wage, job category, working hours, and shiftwork showed that at least one physical burden was strongly associated with pain on all the body parts regardless of psychological job demand or control. Compared with a group with low psychological job demand and no occupational physical burden (a reference group: lowest risk), a group with high psychological job demand and no physical burden did not show an increased risk of pain on any of the body regions. Among those working in jobs with at least one physical burden, the odds ratios were significantly higher compared with the reference group. However, no RERI greater than zero was observed in the analyses with psychological job demand (Table 3).

**TABLE 3:** Logistic regression analyses and relative excess risk due to interaction (RERI) between psychological job demand and occupational physical burdens on musculoskeletal pain in three body regions

Occupational physical burdens	Odds ratio [95% Confidence Interval]*		RERI
	Low psychological demand	High psychological demand	
Back pain			
No	Ref	0.83 [0.71‐0.97]	0.04 [−0.21‐0.29]
At least one	1.85 [1.70‐2.02]	1.69 [1.53‐1.86]	
Pain on neck and upper extremity			
No	Ref	1.03 [0.92‐1.15]	0.02 [−0.17‐0.21]
At least one	2.05 [1.90‐2.20]	2.13 [1.97‐2.29]	
Pain on lower extremity			
No	Ref	1.04 [0.91‐1.18]	0.03 [−0.20‐0.26]
At least one	1.84 [1.70‐2.00]	1.95 [1.79‐2.12]	

* All logistic regression models were adjusted for sex, age, education level, monthly wage, job category, weekly working hours, and shiftwork.

In similar adjusted logistic regression models with an independent variable of job control, compared with a group with high job control and no occupational physical burden (a reference group: lowest risk), a group with low job control and no physical burden also did not show increased risk of pain on all the body parts. However, there was a difference between a group with high job control and at least one physical burden (OR 1.79; 95% CI 1.66‐1.93) and a group with low job control and at least one physical burden (OR 2.44; 95% CI 2.24‐2.66) for pain on neck and upper extremity. The RERI was 0.35 (95% CI 0.19‐0.51). Similar patterns were observed when examining lower0 extremity pain as the outcome. The RERI for pain on lower extremity was 0.26 [0.07‐0.45] (Table 4).

**TABLE 4:** Logistic regression analyses and relative excess risk due to interaction (RERI) between job control and occupational physical burdens on musculoskeletal pain in three body regions

Occupational physical burdens	Odds ratio [95% Confidence Interval]		RERI
	High job control	Low job control	
Back pain			
No	Ref	0.93 [0.82‐1.05]	0.19 [−0.03‐0.41]
At least one	1.72 [1.57‐1.89]	1.90 [1.71‐2.11]	
Pain on neck and upper extremity			
No	Ref	0.96 [0.87‐1.06]	0.35 [0.19‐0.51]
At least one	1.79 [1.66‐1.93]	2.44 [2.24‐2.66]	
Pain on lower extremity			
No	Ref	1.00 [0.90‐1.12]	0.26 [0.07‐0.45]
At least one	1.71 [1.57‐1.86]	2.15 [1.95‐2.37]	

^a^ All logistic regression models were adjusted for sex, age, education level, monthly wage, job category, weekly working hours, and shiftwork.

The same models conducted on waged workers only (sensitivity analyses) showed greater estimates of the main results. In the waged workers, at least one occupational physical burden showed strong associations of pain on all body parts. However, RERI was not increased in the analyses for psychological job demand (Table S1). Low job control was associated with increased ORs for pain on neck and upper extremity/lower extremity. The OR of MSP was greatest in neck and upper extremity pain in the low control and at least one physical factor group (OR 3.10, 95% CI 2.76‐3.48). The ORs and RERIs were greater than the values obtained from the original analyses (Table S2).

## 4 DISCUSSION

This study revealed significant interactions between occupational physical burdens and psychological job demand or control). Psychological job demand or control was not associated with MSP alone; however, job control exacerbated MSP by interaction with occupational physical burdens. The extents of both associations and interactions were greater among waged workers only compared to all types of workers, including employers, self‐employers, and unpaid family workers.

The effects of psychological factors on the development and prevalence of musculoskeletal disorders have been investigated in recent years. Studies using the KWCS found that long‐time work^14^ and work‐life imbalance^15^ were associated with musculoskeletal disorders; job satisfaction was statistically significantly related to musculoskeletal symptoms in office workers, sales workers, and service workers engaged in emotional labor in the 4th KWCS^7^; however, there was no association between job control and any type of pain. Similarly, our initial analysis found that low job control alone had little association with MSP.

Nevertheless, the interaction of low job control and occupational physical burdens was associated with MSP of the neck and upper extremity as well as the lower extremity with significant ORs and significant RERI values. It is noteworthy that the interaction was highest on neck and upper extremity pain. The results are concordant with findings in previous studies, which showed that poor psychological work environment (particularly low job satisfaction and poor social support) and MSP tended to have the highest associations with upper extremity pain.^16^, ^17^, ^18^, ^19^

High work stressors have been found to be associated with musculoskeletal pain. Low control, especially in the workplace, can increase the activity of the sympathetic‐adrenal medullary system, which appears to play an important role in the development of musculoskeletal pain. In a Swiss study, time control was suggested as a risk factor for low‐back pain among nurses beyond the influence of physical workload.^20^ Therefore, prolonged activation of the sympathetic‐adrenal medullary system and inadequate recovery during work may indicate a risk for worsening musculoskeletal pain.^21^

Previously reported studies have examined the relationship between occupational physical burdens and psychological factors in patients with carpal tunnel syndrome. Harris‐Adamson et al showed that the high job strain group (Karasek's job stress model) had increased risk of carpal tunnel syndrome, and social support lowered this risk^22^; in addition, they found an increased risk of carpal tunnel syndrome according to occupational physical burdens in the high job strain group.^23^ When they performed a causal diagram to examine the relationship between occupational physical burdens and psychological factors,^24^ the results indicate that physical and psychological factors act independently as the causes of carpal tunnel syndrome. However, the causal diagram used in those studies is based on the assumption of the causal relationship between variables; therefore, unless a proper stratified analysis is performed, interactions between variables may not be identified. Indeed, those studies did not conduct the stratified analysis to assess the interaction; thus, the causal relationship is still unclear. Notably, our present findings are consistent with those in previous studies, which showed an interaction between psychosocial factors and occupational physical burden on the prevalence of musculoskeletal disorders among workers in New Zealand^25^ and dentists in Malaysia.^26^

The strength of the current study is that it used a large, nationally representative sample including various types of occupations. However, this study has several limitations. First, the study design was cross‐sectional and potential reversed causation could not be ruled out. In the current study, physical symptoms are the dependent variable but assessed for the 12‐month period in advance to the survey. Therefore, the problem arises if physical symptoms might induce employees to rate working conditions as more adverse. Second, all measures are self‐report and therefore common method bias is likely. Moreover, musculoskeletal disorders were assessed using questionnaires regarding presence and absence of symptoms in three parts of a body, so misclassification might occur. Third, it is possible that symptoms caused by problems other than musculoskeletal disorders might be misidentified as musculoskeletal disorders. Therefore, more detailed and structural analyses are needed.

Despite these limitations, this study demonstrates the interactive relationship between occupational physical burdens and low job control on MSP. Our findings suggest two perspectives of practical implications: ergonomic and psychosocial work design. The results support an ergonomic design of work and related measures/activities in order to prevent musculoskeletal disorders. However, our results also suggest that positive effects of such measures addressing physical risk factors in preventing musculoskeletal disorders are more likely to be successful under favorable psychosocial situations. Thus, a combined approach is needed.

## DISCLOSURE

*Approval of the research protocol:* Ethics approval for the present study was not required because this was a retrospective analysis of national surveillance data that are free of personally identifiable information. *Informed consent:* All participants signed a consent form, and anonymity and confidentiality were assured. *Registry and the registration no. of the study/trial:* N/A. *Animal studies:* N/A. *Conflict of interest:* N/A.

## CONFLICT OF INTEREST

Authors declare no conflicts of interest.

## AUTHOR CONTRIBUTIONS

JL conceived of and designed the study. JL conducted statistical analyses. MYK and JL drafted and revised the manuscript. MYK, JL, HRK, and DWL interpreted the data and provided critical revision of the manuscript. MYK supervised the study.

## Supporting information

Table S1‐S2
