# CORRIGENDUM

**DOI:** 10.1002/1348-9585.12324

**Published:** 2022-04-05

In Lee J,^1^ the following error was published in the section text.

In the second paragraph of the "Definition of variables" section on page 2, there is the following sentence:

"In response, the numbers of the relevant items were regarded as the score for the four questions and were summed up into one value. The participants who had lower than median values (10 points) were classified as the low job control group."

The sentence was incorrect and should have read:

"In response, the numbers of the relevant items were regarded as the score for the three questions and were summed up into one value. The participants who had lower than median values (8 points) were classified as the low job control group."

The authors apologize for this error.
