# Supplementary material for: Interaction between occupational physical burdens and low job control on musculoskeletal pain: Analysis of the 5th Korean Working Environment Survey
Source: J Occup Health. 2021 Jul 1;63(1):e12244. doi: 10.1002/1348-9585.12244 (PMC8249184; doi:10.1002/1348-9585.12244)
Supplement: Supplementary file 1 — Table S1‐S2 [file JOH2-63-e12244-s001.docx]

sUPPLEMENTARY TABLE 1 Logistic regression analyses and relative excess risk due to interaction (RERI) between occupational physical burdens and psychological demand in three body parts of musculoskeletal pain only among waged workers

|  |  | **Odds ratio [95% Confidence Interval]** | | **RERI** |
| --- | --- | --- | --- | --- |
|  | **Occupational physical burdens** | **Low psychological demand** | **High psychological demand** |  |
| **<Back pain>** | |  |  |  |
|  | No | Ref | 0.99 [0.80-1.20] | -0.02 [-0.37-0.33] |
|  | At least one | 1.93 [1.70-2.19] | 1.89 [1.66-2.16] |  |
| **<Pain on neck and upper extremity>** | |  |  |  |
|  | No | Ref | 1.13 [0.98-1.29] | 0.05 [-0.20-0.31] |
|  | At least one | 2.15 [1.95-2.37] | 2.43 [2.20-2.68] |  |
| **<Pain on lower extremity>** | |  |  |  |
|  | No | Ref | 1.07 [0.90-1.26] | 0.08 [-0.23-0.40] |
|  | At least one | 1.87 [1.67-2.10] | 2.08 [1.86-2.32] |  |

^*^All the results in the table were from adjusted models for sex, age, education level, monthly wage, job category, weekly working hours, and shiftwork.

sUPPLEMENTARY TABLE 2 Logistic regression analyses and relative excess risk due to interaction (RERI) between occupational physical burdens and job control in three body parts of musculoskeletal pain only among waged workers

|  |  | **Odds ratio [95% Confidence Interval]** | | **RERI** |
| --- | --- | --- | --- | --- |
|  | **Occupational physical burdens** | **High job control** | **Low job control** |  |
| **<Back pain>** | |  |  |  |
|  | No | Ref | 1.12 [0.94-1.32] | 0.37 [0.06-0.68] |
|  | At least one | 1.73 [1.47-2.03] | 2.35 [2.02-2.73] |  |
| **<Pain on neck and upper extremity>** | |  |  |  |
|  | No | Ref | 1.21 [1.06-1.37] | 0.48 [0.26-0.70] |
|  | At least one | 1.91 [1.70-2.14] | 3.10 [2.76-3.48] |  |
| **<Pain on lower extremity>** | |  |  |  |
|  | No | Ref | 1.25 [1.08-1.45] | 0.44 [0.16-0.72] |
|  | At least one | 1.73 [1.51-1.99] | 2.70 [2.37-3.09] |  |

^*^All the results in the table were from adjusted models for sex, age, education level, monthly wage, job category, weekly working hours, and shiftwork.
